# Phenotype–Genotype Discordance in Antimicrobial Resistance of *Acinetobacter baumannii*: Implications for Diagnostics and Surveillance

**DOI:** 10.3390/pathogens15040381

**Published:** 2026-04-02

**Authors:** Nazgul Sutimbekova, Nelya Bissenova, Marat Dusmagambetov, Ulbossyn Saltabayeva, Aigul Utegenova, Gulmira Smanova, Alfiya Igissenova, Farida Rakhimzhanova, Nurgul Askarova, Gulbanu Duissebekova, Ayaz Yktiyarov, Evgeni Sokurenko

**Affiliations:** 1 Department of Microbiology and Virology, Astana Medical University, Astana 010000, Kazakhstan; sutimbekova.n@amu.kz (N.S.); dusmagambetov.m@amu.kz (M.D.); dusebekova.g@amu.kz (G.D.); yktiyarov.a@amu.kz (A.Y.); 2 Microbiological Laboratory, National Scientific Medical Center, Astana 010000, Kazakhstan; n.bissenova@nnmc.kz; 3 Department of Nursing, Astana Medical University, Astana 010000, Kazakhstan; 4 Department of Medical Genetics and Molecular Biology, Astana Medical University, Astana 010000, Kazakhstan; smanova.g@amu.kz; 5 Department of Microbiology and Virology, Asfendiyarov Kazakh National Medical University, Almaty 050012, Kazakhstan; igisenova.a@kaznmu.kz; 6 Department of Microbiology, Semey Medical University, Semey 071400, Kazakhstan; farida.rakhimzhanova@smu.edu.kz; 7 Department of Normal Physiology, Astana Medical University, Astana 010000, Kazakhstan; askarova.nu@amu.kz; 8 Department of Microbiology, School of Medicine, University of Washington, Seattle, WA 98195, USA; evs@uw.edu

**Keywords:** *Acinetobacter baumannii*, antimicrobial resistance, carbapenem resistance, genotype–phenotype correlation, antimicrobial susceptibility testing, carbapenemases, aminoglycoside-modifying enzymes, whole-genome sequencing

## Abstract

*Acinetobacter baumannii* has emerged as one of the most challenging opportunistic pathogens in modern healthcare due to its remarkable ability to acquire and disseminate antimicrobial resistance determinants. Carbapenem-resistant *A. baumannii* (CRAB) is now recognized by the World Health Organization as a critical priority pathogen, highlighting the urgent need for improved diagnostic, surveillance, and therapeutic strategies. This review synthesizes current evidence on the relationship between phenotypic antimicrobial susceptibility patterns and underlying genetic determinants of resistance in *A. baumannii*. A structured literature search was conducted across major biomedical databases (PubMed/MEDLINE, Scopus, Web of Science, and the Cochrane Library), supplemented by citation tracking and relevant institutional sources, focusing on studies published between 2016 and 2026. The analysis integrates findings from studies examining phenotypic antimicrobial susceptibility testing (AST) with molecular and genomic investigations of resistance mechanisms, including carbapenemases, aminoglycoside-modifying enzymes, efflux pumps, and resistance-associated genomic islands. Particular attention is given to the complex and sometimes discordant relationship between genotype and phenotype, where the presence of resistance genes does not always translate directly into phenotypic resistance due to regulatory mechanisms, gene expression variability, and genomic context. The review further discusses methodological differences in AST standards and genomic prediction approaches that may contribute to variability across studies. Collectively, the evidence supports a multidimensional interpretation of antimicrobial resistance in *A. baumannii*, emphasizing the necessity of integrating phenotypic and genotypic data for accurate diagnostics, surveillance, and clinical decision-making. This integrated perspective may contribute to improved understanding of resistance evolution and support the development of more effective strategies for managing multidrug-resistant *A. baumannii* infections.

## 1. Introduction

Antimicrobial resistance (AMR) is widely recognized as a major threat to global health systems, undermining the effectiveness of essential antimicrobial therapies and increasing the burden of hospital-acquired infections (HAIs) [1]. Among Gram-negative pathogens, *Acinetobacter baumannii* has become emblematic of the AMR crisis due to its exceptional capacity to persist in healthcare environments, colonize vulnerable patients, and acquire multidrug resistance through both chromosomal adaptation and horizontal gene transfer [2,3]. Clinically, *A. baumannii* is strongly associated with ventilator-associated pneumonia, bloodstream infections, complicated wound infections, and device-associated infections, particularly in intensive care settings where antimicrobial selection pressure and invasive procedures amplify transmission risk [2,4]. Reflecting this urgency, the World Health Organization (WHO) has consistently listed carbapenem-resistant *A. baumannii* among the highest-priority bacterial threats, emphasizing the need for improved surveillance, infection control, and development of effective treatment strategies [5,6].

The success of *A. baumannii* as a nosocomial pathogen is driven by a convergence of ecological fitness and resistance biology. This organism tolerates desiccation and disinfectant stress better than many other non-fermenters, facilitating long-term environmental persistence and repeated opportunities for patient-to-patient spread [2,3]. At the same time, the species demonstrates a remarkable “resistome plasticity,” allowing rapid adaptation under antimicrobial pressure through acquisition of resistance determinants and modulation of envelope permeability, efflux, and enzymatic inactivation pathways [3,7]. Importantly, resistance and virulence are not independent axes; emerging evidence indicates that resistance mechanisms can reshape bacterial fitness landscapes and alter pathogenic potential, complicating predictions of clinical outcomes based solely on susceptibility categories [4,8].

The primary resistance determinants driving this adaptive landscape include OXA-type carbapenemases (class D β-lactamases, notably *blaOXA-23*, *blaOXA-40*, *blaOXA-51*), metallo-β-lactamases (class B, including *blaVIM* and *blaNDM*), extended-spectrum β-lactamases (class A, e.g., *blaTEM*), and aminoglycoside-modifying enzymes (*AME*s, including *aacA4* and *aacC1*). The molecular classification, mechanisms, and clinical significance of these resistance classes are reviewed in detail in Section 3.3.

Phenotypic antimicrobial susceptibility testing and molecular detection of resistance genes therefore provide complementary perspectives on antimicrobial resistance in *A. baumannii* epidemiological surveillance. This phenotype–genotype discordance represents a critical knowledge gap: existing literature has documented instances of both false-susceptibility (resistance genes present but phenotype susceptible) and false-resistance (phenotypic resistance without canonical resistance genes), yet the prevalence, mechanistic basis, and clinical impact of these discordant outcomes remain incompletely characterized. Consequently, a systematic synthesis of current evidence linking phenotypic susceptibility testing with genotypic resistance determinants is necessary to clarify how these complementary approaches can be interpreted in routine clinical microbiology practice, and to identify where integrated phenotypic–genotypic frameworks are most needed.

The aim of this review is to critically synthesize current evidence on phenotypic and genotypic approaches to antimicrobial resistance assessment in *Acinetobacter baumannii*, with particular emphasis on the interpretation of key resistance genes (*bla*OXA-23, *bla*OXA-51, *bla*OXA-40, *bla*TEM, *bla*VIM, *bla*SPM, *aacA4*, *aacC1*) in relation to clinical AST results, and to highlight how integrated phenotypic–genotypic interpretation can improve diagnostic accuracy, antimicrobial stewardship decisions, and epidemiological surveillance of multidrug-resistant *A. baumannii* in healthcare settings.

## 2. Materials and Methods

Study Design and Review Framework. This study was conducted as a narrative review with structured methodological elements and a transparent literature selection process designed to synthesize current evidence on phenotypic and genotypic approaches to antimicrobial resistance detection in *Acinetobacter baumannii*. The review framework was designed to integrate data derived from phenotypic AST and molecular genetic analyses, with particular emphasis on clinically relevant resistance determinants and their interpretation in hospital settings.

The methodological approach combined (i) a systematic and transparent literature identification and selection process, and (ii) a structured analytical description of phenotypic and genotypic resistance detection methods commonly applied in clinical microbiology laboratories. This approach allows positioning of the reviewed evidence within real-world diagnostic and surveillance workflows.

Literature Search Strategy. A comprehensive literature search was performed using the PubMed/MEDLINE, Scopus, Web of Science, and Cochrane Library databases. The primary search term was “*Acinetobacter baumannii*”, which initially yielded more than 16,000 records. To ensure contemporary relevance, the publication period was restricted to January 2016 through January 2026, resulting in over 11,000 records.

The search strategy was further refined using combinations of keywords and controlled vocabulary terms related to antimicrobial resistance and diagnostic approaches, including “antimicrobial resistance”, “phenotypic susceptibility testing”, “disk diffusion”, “EUCAST”, “carbapenem resistance”, “OXA-type carbapenemases”, “metallo-β-lactamases”, “aminoglycoside resistance”, “PCR”, “resistance genes”, and “phenotype-genotype correlation”.

**Figure 1:**
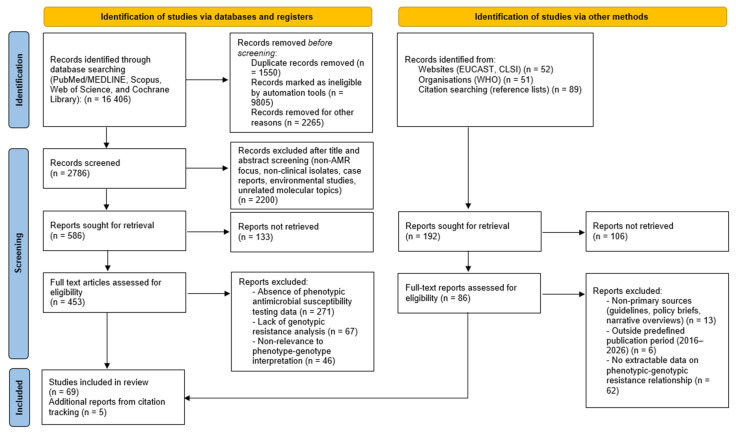
PRISMA-like flow diagram of literature identification, screening, and study selection for the review.

Additional records were identified through manual screening of reference lists and inclusion of official international documents and guidelines (e.g., WHO and EUCAST). The overall process of literature identification, screening, eligibility assessment, and inclusion is summarized in a PRISMA-like flow diagram (Figure 1).

Study Selection and Eligibility Criteria. After removal of duplicate records, titles and abstracts were screened for relevance. Studies were eligible for full-text assessment if they met the following criteria:

- Focused on *Acinetobacter baumannii* clinical isolates;
- Reported phenotypic AST results using standardized methodologies (e.g., disk diffusion or MIC-based systems);
- Included molecular detection of antimicrobial resistance determinants;
- Addressed clinical, epidemiological, or methodological aspects of resistance interpretation.

Studies were excluded if they focused exclusively on non-clinical or environmental isolates, lacked methodological detail regarding resistance detection, or did not contribute to understanding phenotypic–genotypic relationships. Following full-text assessments, 74 studies were included in the qualitative synthesis (69 identified through database searching and 5 additional reports identified through citation tracking) (Figure 1).

Phenotypic Antimicrobial Susceptibility Testing. Phenotypic resistance assessment in *A. baumannii* is primarily based on AST, performed using disk diffusion methods and automated susceptibility testing systems. In the reviewed studies, disk diffusion testing was typically conducted on Mueller–Hinton agar with interpretation of inhibition zone diameters according to European Committee on Antimicrobial Susceptibility Testing (EUCAST) breakpoints. Automated systems, including MIC-based platforms, were also commonly employed to categorize isolates as *susceptible* (S), *intermediate* (I), or *resistant* (R) according to the applicable breakpoint criteria.

Phenotypic AST provides an integrative assessment of expressed resistance mechanisms but may be influenced by methodological variability, breakpoint revisions, inoculum effects, and regulatory control of gene expression.

Genotypic Detection of Resistance Determinants. Genotypic resistance detection was predominantly performed using polymerase chain reaction (PCR) assays, with sequencing applied in selected studies for confirmation. The review focused on resistance genes frequently reported in clinical *A. baumannii* isolates, including OXA-type carbapenemase genes (*blaOXA-23*, *blaOXA-40*, *blaOXA-51*), β-lactamase genes (*blaTEM*), metallo-β-lactamase genes (*blaVIM*, *blaSPM*), and aminoglycoside-modifying enzyme genes (*aacA4*, *aacC1*).

Several studies also examined the genetic context of these determinants, such as insertion sequences, integrons, and mobile genetic elements, which modulate gene expression and dissemination and contribute to variability in phenotypic resistance profiles.

Data Synthesis and Analysis. Data from included studies were synthesized qualitatively. Particular attention was paid to reported concordance or discordance between phenotypic AST results and detected resistance genes, methodological factors influencing resistance interpretation, and clinical implications for antimicrobial therapy and infection control. Due to heterogeneity in study design and reporting, no quantitative meta-analysis was performed.

Ethical Considerations. Ethical review and approval were not required for this study because it is based exclusively on the analysis and synthesis of previously published literature.

## 3. Results

### 3.1. Phenotypic and Genotypic Antimicrobial Resistance in Acinetobacter baumannii

Based on the PRISMA-like selection process (Figure 1), a total of 74 publications were included for qualitative synthesis, providing a comprehensive overview of antimicrobial resistance (AMR) in geographic regions. In terms of geographic distribution, the majority of included studies originated from Asia (approximately 40%, predominantly China, India, South Korea, and Iran), followed by Europe (~25%, including Turkey, Poland, Spain, and Italy), the Middle East (~15%), and the Americas (~10%), with the remaining studies representing multi-country surveillance or African settings. This distribution reflects both the burden of CRAB in these regions and publication patterns in the indexed literature, and should be considered when extrapolating findings to other geographic contexts. Numerically, among the 74 included publications: 58 studies (78%) reported carbapenem resistance rates exceeding 50% in their clinical isolate collections; 46 studies (62%) described MDR or XDR phenotypes as the dominant pattern; 39 studies (53%) provided concurrent genotypic data linking resistance phenotypes to specific determinants; and 31 studies (42%) employed WGS or comprehensive molecular typing approaches.

Phenotypic AST data reported across studies demonstrate a high prevalence of multidrug-resistant (MDR) and extensively drug-resistant (XDR) phenotypes. Resistance to β-lactam antibiotics, including extended-spectrum cephalosporins and carbapenems, represents the dominant phenotypic pattern in contemporary *A. baumannii* populations [1,6,9,10]. Numerous studies document carbapenem non-susceptibility rates exceeding 50–70% in hospital-derived isolates, with even higher rates reported in outbreak-associated or ICU-specific cohorts [2,7,11].

Aminoglycoside resistance is another frequently reported phenotypic feature, and often co-occurs with carbapenem resistance, further limiting therapeutic options [8,9,12]. High resistance rates to gentamicin, amikacin, and tobramycin have been observed in multiple clinical settings, particularly among MDR and XDR isolates [8,12]. However, several studies emphasize that aminoglycoside resistance patterns may vary independently of carbapenem resistance status, highlighting the importance of comprehensive AST rather than reliance on surrogate markers [9,12].

Despite the overall dominance of resistant phenotypes, substantial heterogeneity in susceptibility profiles is observed across regions and time periods. This variability is influenced by local antimicrobial stewardship practices, infection control measures, circulating clonal lineages, and methodological factors such as AST platforms and breakpoint versions [6,10,13]. Importantly, phenotypic AST provides a functional assessment of expressed resistance but does not elucidate the underlying molecular mechanisms responsible for the observed patterns.

Genotypic investigations summarized in the reviewed literature reveal that carbapenem resistance in *A. baumannii* is most commonly associated with OXA-type carbapenemases, while aminoglycoside resistance is frequently mediated by aminoglycoside-modifying enzymes [1,7,8,9,10,11,12,14]. However, the presence of resistance genes does not always translate into uniform phenotypic expression. Several studies report discordance between detected resistance determinants and phenotypic susceptibility results, underscoring the role of gene expression levels, regulatory elements, and additional mechanisms such as efflux systems and membrane permeability changes [1,13,15,16,17].

Overall, the reviewed evidence supports the concept that phenotypic and genotypic resistance profiles in *A. baumannii* are complementary rather than interchangeable. While phenotypic AST remains indispensable for clinical decision-making, integrated interpretation alongside molecular data provides a more accurate and informative framework for AMR surveillance, epidemiological analysis, and optimization of antimicrobial therapy [1,13,18].

### 3.2. Phenotypic Antimicrobial Resistance Patterns (Carbapenems, Aminoglycosides)

Phenotypic carbapenem non-susceptibility is the dominant and most clinically consequential resistance pattern reported for *Acinetobacter baumannii* in contemporary hospital microbiology, especially in ICU-associated cohorts [1,6,10,19]. Across multiple surveillance-oriented and hospital-based studies, carbapenem-resistant *A. baumannii* (CRAB) is repeatedly linked to restricted effective empirical options and delayed initiation of active therapy, which—at the population level—translates into worse outcomes compared with carbapenem-susceptible infections [20,21]. Meta-analytic synthesis supports a consistent association between CRAB and adverse clinical endpoints (including higher mortality and greater severity), indicating that the phenotypic label “carbapenem-resistant” is not merely a laboratory classification but a clinically informative stratifier of risk [21]. Regional systematic reviews further demonstrate that the burden and pooled prevalence of carbapenem resistance can be substantial and heterogeneous across settings, reflecting differences in infection control, antimicrobial consumption, and circulating lineages [22,23]. This heterogeneity is also visible in global narratives positioning *A. baumannii* among the highest priority pathogens due to the convergence of persistence, limited therapy, and resistance amplification in high-intensity care environments [20,24].

From a laboratory perspective, a key interpretive constraint in phenotypic CRAB reporting is the strong dependence on (i) the AST platform (disk diffusion vs. MIC-based methods), (ii) the chosen interpretive standard (EUCAST vs. CLSI), and (iii) breakpoint versioning [6,25,26]. EUCAST explicitly frames MIC as the basis of categorization and provides standardized disk diffusion methodology and QC expectations that are designed to improve cross-laboratory comparability [25]. CLSI similarly provides performance standards and interpretive criteria, but laboratories using different standards or transitioning between editions can observe categorical shifts for the same isolate–drug pair, particularly around breakpoints where small technical or biological variations produce S/I/R changes [26]. Consequently, phenotypic CRAB prevalence should be interpreted as method-bound: apparent increases or decreases across time or sites can reflect true epidemiology, but they can also be partially driven by changes in interpretive rules, testing materials, or QC adherence [6,25,26,27,28]. For manuscript rigor, this is important because it justifies why many high-quality studies report both categorical results and the underlying quantitative readouts (zone diameters and/or MIC distributions), enabling reproducibility and secondary synthesis.

Phenotypic carbapenem resistance in *A. baumannii* is also rarely an isolated phenotype. Reviews integrating phenotypic datasets consistently show that carbapenem non-susceptibility frequently co-segregates with non-susceptibility to third-/fourth-generation cephalosporins and often with fluoroquinolone resistance, forming MDR/XDR profiles that markedly constrain therapeutic choices [1,9,10,29]. However, the literature also notes that the degree of carbapenem resistance (low-level vs. high-level MICs/zone reductions) can vary between cohorts, and this variation is mechanistically meaningful because it may reflect differences in the dominant resistance pathways and their expression intensity (e.g., promoter-driven overexpression vs. additional permeability/efflux contributions) [13,17,29]. Even within phenotypic-only reporting, the repeated observation of “broad β-lactam resistance + carbapenem resistance” supports the inference of multiple concurrent mechanisms rather than a single determinant [1,13].

Aminoglycoside resistance constitutes the second major phenotypic axis shaping *A. baumannii* treatment limitations. Multiple clinical studies report high rates of non-susceptibility to gentamicin, amikacin, and/or tobramycin, particularly in MDR/XDR strata and ICU-derived isolates [8,9,12]. Importantly, aminoglycoside resistance frequently—but not invariably—tracks with CRAB. This partial coupling has two practical implications. First, aminoglycosides cannot be assumed effective “salvage partners” in CRAB infections without isolate-specific AST confirmation [9,12]. Second, the observed discordant phenotypes (e.g., CRAB with retained aminoglycoside susceptibility, or carbapenem-susceptible isolates with aminoglycoside resistance) reinforce that resistance phenotypes evolve through modular acquisition/selection processes rather than a single uniform pathway [8,12,13]. Mechanistic reviews of aminoglycoside-modifying enzymes emphasize that phenotypic aminoglycoside resistance is often driven by enzymatic inactivation, but clinical datasets show that the phenotypic outcome depends on which enzyme set is present, the substrate profile, and co-existing resistance architecture [23]. This aligns with ICU-focused reports where aminoglycoside resistance genes and high phenotypic resistance rates co-occur, consistent with strong selection pressure and mobile element-mediated spread [12,27,28].

An additional, underappreciated interpretive layer is that phenotypic aminoglycoside categorization can be sensitive to testing conditions and interpretive criteria. While aminoglycoside AST is generally robust, borderline MIC/zone cases and mixed populations can still produce variability, especially when laboratories rely on a single platform without confirmatory testing for critical clinical decisions [25,26]. Therefore, when synthesizing phenotypic aminoglycoside patterns across studies, a high-quality approach is to prioritize datasets that explicitly state the testing standard and QC framework and to interpret inter-study differences in light of method comparability [6,25,26].

In sum, the phenotypic evidence base supports three stable conclusions: (i) carbapenem resistance is the central phenotype defining contemporary high-risk *A. baumannii* epidemiology and is consistently associated with adverse outcomes [21]; (ii) aminoglycoside resistance is highly prevalent in severe resistance strata and further narrows combination therapy feasibility [8,9,12,28]; and (iii) reported resistance rates are meaningfully influenced by AST methodology and breakpoint frameworks, mandating transparent reporting and cautious cross-study comparisons [6,25,26,27,28,29]. These phenotypic patterns provide the necessary foundation for the subsequent mechanistic synthesis of genotypic determinants and phenotype–genotype concordance.

### 3.3. Genotypic Determinants Associated with Carbapenem and Aminoglycoside Resistance

This section focuses on the genotypic determinants of carbapenem and aminoglycoside resistance, as these two antibiotic classes represent the most clinically consequential axes of resistance in *A. baumannii* and were consistently represented across the included literature. Resistance to other antibiotic classes—including fluoroquinolones, tetracyclines, colistin, and trimethoprim-sulfamethoxazole—is recognized as clinically important but falls outside the defined scope of this review, which was intentionally designed to provide a focused synthesis of phenotype–genotype relationships for the most frequently studied and therapeutically critical determinants. The inclusion criteria prioritized studies reporting concurrent phenotypic AST and molecular or genomic data for carbapenem and aminoglycoside resistance, consistent with the review’s objective of evaluating concordance between these two analytical approaches.

β-lactam resistance in *A. baumannii* also involves extended-spectrum β-lactamases (ESBLs) such as TEM-type enzymes (e.g., *blaTEM*), which can contribute to resistance profiles when combined with permeability changes and other β-lactamases [13,19]. Meanwhile, aminoglycoside resistance is commonly mediated by aminoglycoside-modifying enzymes (AMEs), including acetyltransferases such as *aacA4* and gentamicin-modifying determinants such as *aacC1*, which reduce drug activity and can contribute to multidrug-resistant (MDR) phenotypes when present alongside β-lactam resistance determinants [15,16,17]. These resistance genes frequently cluster with additional determinants on plasmids or integrons, reinforcing the concept that MDR in *A. baumannii* is often a “genetic package deal”, not a single-gene event [3,14,15].

Beyond OXA-type enzymes, metallo-β-lactamases (MBLs) represent an additional layer of threat due to their broad hydrolytic activity against β-lactams, including carbapenems. Genes such as *blaVIM* (and less commonly *blaSPM* in many settings) have been reported as contributors to high-level resistance, often carried on mobile genetic elements that facilitate dissemination across taxa and hospital ecosystems [11,12,13,14]. The clinical significance of MBLs extends beyond resistance: MBL producers frequently exhibit co-resistance to multiple classes, leading to severely constrained treatment options and forcing reliance on older agents with toxicity concerns [10,13]. While not all local populations display MBLs at high prevalence, their intermittent emergence, outbreak association, and mobility justify their inclusion in targeted molecular panels for surveillance [11,12,13,14].

Beta-lactamases are classified according to the Ambler molecular classification system into four classes based on their amino acid sequences and active-site residues. Class A enzymes (e.g., TEM-type ESBLs, KPC carbapenemases) utilize a serine residue at the active site and include many clinically important extended-spectrum and carbapenem-hydrolyzing enzymes. Class B enzymes are metallo-β-lactamases (MBLs), which require zinc ions for catalytic activity and include VIM, NDM, and IMP variants capable of hydrolyzing virtually all β-lactams, including carbapenems, but not monobactams. Class C enzymes (AmpC-type cephalosporinases) are serine-based enzymes that confer resistance to cephalosporins and are generally not inhibited by classic β-lactamase inhibitors. Class D enzymes (OXA-type oxacillinases) are also serine-based and encompass the clinically dominant carbapenemases in *A. baumannii*, including the OXA-23-like, OXA-24/40-like, and intrinsic OXA-51-like subgroups. Understanding this classification is essential for interpreting resistance determinants, as each class carries distinct substrate profiles, inhibitor susceptibilities, and epidemiological characteristics [12,14,30].

Carbapenem resistance in *A. baumannii* is of particular clinical concern because it compromises one of the most important therapeutic backbones for severe Gram-negative infections. The dominant mechanisms of carbapenem resistance are frequently linked to OXA-type carbapenemases (class D β-lactamases), with blaOXA-23 representing one of the most globally disseminated acquired carbapenemase genes in *A. baumannii* [12,30]. The genetic context is critical: insertion sequences such as IS*Aba1* can enhance expression of OXA enzymes and thereby increase resistance levels, demonstrating that detection of a gene may not fully describe the magnitude of the phenotype without considering upstream regulatory architecture [24]. In parallel, blaOXA-40 (often discussed as OXA-24/40-like) has been implicated in carbapenem resistance across multiple regions, further highlighting the diversity of OXA-mediated resistance [9,12]. In contrast, blaOXA-51-like genes are typically intrinsic to *A. baumannii* and are frequently used as species-associated markers; however, their contribution to carbapenem resistance depends strongly on expression and mobilization events, which again emphasizes that “gene presence” and “resistance expression” are not always synonymous [2,12,24].

Genotypic analyses across the reviewed studies indicate that carbapenem resistance in *Acinetobacter baumannii* is primarily driven by class D β-lactamases (OXA-type carbapenemases), frequently modulated by insertion sequence-mediated regulatory effects [1,3,7,10,13,17]. The intrinsic *bla*OXA-51-like gene, universally present in *A. baumannii*, functions as a species-specific marker but typically requires upstream promoter activation to significantly influence carbapenem susceptibility [1,13]. High-level carbapenem resistance is most consistently associated with acquired oxacillinases, particularly *bla*OXA-23-like and *bla*OXA-24/40-like determinants, which dominate in clinical carbapenem-resistant *A. baumannii* (CRAB) populations worldwide [2,7,13,14].

A recurrent genetic feature underlying carbapenem resistance involves insertion sequences such as IS*Aba1*, which enhance transcription of adjacent β-lactamase genes. Contemporary genomic investigations confirm that IS*Aba1*-mediated amplification and promoter activity represent central mechanisms facilitating the phenotypic expression of carbapenem resistance [31,32]. These regulatory interactions explain why isolates harboring similar carbapenemase genes may exhibit variable phenotypic resistance levels, emphasizing that gene presence alone is insufficient for predicting resistance magnitude [1,13].

Among acquired carbapenemases, *blaOXA-23* remains the most globally disseminated determinant, often embedded within mobile genetic elements and associated with international high-risk clones [13,29]. Recent genomic surveillance studies further reveal the long-term persistence and clonal stability of *bla*OXA-24/40-producing lineages in hospital environments, highlighting the evolutionary success of these genotypes [33]. The epidemiological significance of OXA-24/40 enzymes lies in their capacity to sustain endemic carbapenem resistance even in the absence of large-scale outbreak events.

Although metallo-β-lactamases (MBLs) are less frequently detected in *A. baumannii* than OXA-type enzymes, their clinical relevance is substantial. Systematic analyses demonstrate that MBL-producing *Acinetobacter* isolates, including those carrying *bla*VIM-like genes, are associated with extensive resistance profiles and reduced therapeutic options [34]. The coexistence of MBLs with OXA carbapenemases further contributes to complex resistance architectures and may complicate phenotypic interpretation of β-lactam susceptibility [1,16].

Aminoglycoside resistance in *A. baumannii* is predominantly mediated by genes encoding aminoglycoside-modifying enzymes (AMEs), including acetyltransferases and nucleotidyltransferases [8,12,15]. Genes such as *aacC1* and *aacA4* are widely distributed among clinical isolates and frequently reside within integrons or resistance islands, facilitating horizontal gene transfer [8,12,35]. Recent genomic analyses demonstrate that AME determinants are commonly co-localized with carbapenem resistance genes within composite mobile elements, supporting the concept of co-selection under antimicrobial pressure [28,36].

The structural organization of resistance determinants in *A. baumannii* is strongly influenced by genomic resistance islands (*AbaR*, *AbGRI*), which serve as reservoirs for multidrug resistance genes [13,37,38,39]. Modern genome-scale studies reveal that these elements display considerable diversity in insertion sites and gene content, often correlating with specific clonal lineages [37]. Such findings provide a mechanistic explanation for the stable association between particular epidemic clones and characteristic resistance phenotypes observed in clinical surveillance.

Collectively, current evidence supports a model in which carbapenem and aminoglycoside resistance genotypes evolve through the combined action of acquired β-lactamases, AME genes, insertion sequences, and genomic islands. This multilayered genetic framework explains both the high prevalence of MDR/XDR phenotypes and the observed variability in phenotypic resistance expression [1,13,40].

In routine practice, AMR detection begins with phenotypic antimicrobial susceptibility testing (AST), including disk diffusion and automated MIC-based systems, interpreted using standardized breakpoints such as EUCAST [18]. Phenotypic AST remains indispensable because it directly captures the net effect of all resistance mechanisms expressed under test conditions. However, phenotypic methods can be influenced by inoculum effects, methodological variability, breakpoint changes over time, and the dynamic interplay between resistance determinants and expression levels [18]. Conversely, molecular testing (PCR, sequencing, and increasingly WGS) offers mechanistic resolution by identifying resistance determinants and their genetic contexts, supporting outbreak investigations and predictive modeling [3,7]. Yet, genotype does not always perfectly predict phenotype: gene expression regulation, insertion sequences, promoter strength, gene dosage, epistatic interactions, and unmeasured mechanisms (e.g., efflux modulation, porin alterations) can generate either high concordance or meaningful discordance between phenotypic and genotypic findings [3,7,15,24]. From a clinical and epidemiological perspective, accurate interpretation of these relationships is essential for guiding empiric therapy, optimizing antimicrobial stewardship strategies, and strengthening hospital surveillance systems aimed at early detection of emerging resistance patterns.

### 3.4. Phenotype–Genotype Concordance and Discordance

The relationship between phenotypic antimicrobial susceptibility profiles and underlying genotypic resistance determinants in *Acinetobacter baumannii* is inherently multifactorial and frequently deviates from a strictly deterministic model. Although strong correlations between resistance genes and phenotypic resistance patterns are commonly reported, discordant observations remain a recurrent finding across clinical datasets [1,13,17,40]. This complexity is particularly evident for carbapenems and aminoglycosides, where genotype alone does not uniformly predict antimicrobial susceptibility outcomes.

For carbapenem resistance, acquired oxacillinase genes such as *bla*OXA-23-like and *bla*OXA-24/40-like variants are widely recognized as major contributors to phenotypic resistance [7,13,31,33]. However, multiple studies demonstrate that isolates carrying identical carbapenemase genes may exhibit variable MIC values and heterogeneous resistance phenotypes [31,32,40]. Such variability is largely driven by regulatory rather than structural genetic differences. Insertion sequences, particularly IS*Aba1*, are known to modulate transcriptional activity of adjacent β-lactamase genes, thereby influencing resistance magnitude and producing phenotype–genotype variability [31,32]. Consequently, resistance gene detection alone does not necessarily correspond to uniform phenotypic expression.

Conversely, phenotypic carbapenem resistance has been described in isolates lacking detectable carbapenemase genes [1,13]. These observations indicate the involvement of alternative mechanisms, including efflux pump overexpression, altered membrane permeability, and regulatory adaptations that collectively affect antimicrobial susceptibility [15,41]. Experimental and transcriptomic investigations confirm that resistance-nodulation-division (RND) efflux systems may significantly contribute to carbapenem and multidrug resistance phenotypes independent of carbapenemase production [41,42]. Such findings highlight intrinsic biological contributors to apparent genotype–phenotype discordance.

Aminoglycoside resistance patterns exhibit comparable complexity. Aminoglycoside-modifying enzyme (AME) genes, including *aacC1* and *aacA4*, are strongly associated with resistance phenotypes [8,12,35], yet phenotypic expression varies substantially among isolates [43]. Variability in substrate specificity, gene expression dynamics, and combinatorial resistance mechanisms can produce incomplete or selective resistance profiles, resulting in partial susceptibility despite AME gene presence [12,43]. These patterns reinforce that phenotypic resistance represents a functional outcome influenced by multiple regulatory and biochemical factors.

Methodological considerations further complicate phenotype–genotype interpretation. Variability between antimicrobial susceptibility testing platforms, interpretive standards, and breakpoint systems has been extensively documented [25,26,42]. Differences between EUCAST- and CLSI-based categorization frameworks may alter susceptibility classifications without reflecting underlying biological divergence [42]. Similarly, molecular detection sensitivity and genome-based resistance prediction algorithms may yield discrepancies depending on analytical pipelines and reference databases [44,45,46,47,48].

Recent integrative analyses emphasize that phenotype–genotype discordance may arise from both biological and technical sources [46,47,48]. Genome-based resistance prediction models, while increasingly powerful, remain constrained by the incomplete understanding of regulatory effects, gene expression variability, and novel resistance determinants [45,48]. Therefore, phenotypic and genotypic resistance data in *A. baumannii* should be interpreted as complementary analytical dimensions rather than interchangeable indicators.

The principal scenarios underlying phenotype–genotype concordance and discordance are summarized in Table 1, which integrates mechanistic and methodological explanations supported by the reviewed literature.

Collectively, current evidence supports the interpretation that phenotypic and genotypic resistance data represent complementary analytical dimensions. Integrated frameworks accounting for gene presence, expression regulation, genomic context, and quantitative phenotypic measurements provide the most robust model for understanding antimicrobial resistance behavior in *A. baumannii* [1,13,48].

### 3.5. Distribution and Epidemiological Significance of Key Resistance Genes

The distribution of antimicrobial resistance genes in *Acinetobacter baumannii* populations represents a central determinant of both phenotypic resistance patterns and the broader epidemiological behavior of this pathogen. Across the reviewed literature, carbapenem resistance is consistently associated with the dissemination of acquired OXA-type β-lactamases, particularly *bla*OXA-23-like and *bla*OXA-24/40-like determinants, which exhibit marked geographic variability yet remain globally dominant [1,7,13,31,33].

The intrinsic *bla*OXA-51-like gene, detected in nearly all *A. baumannii* isolates, functions primarily as a taxonomic marker rather than a direct predictor of carbapenem resistance [1,13]. However, its epidemiological significance lies in its capacity for insertion sequence-mediated overexpression, a mechanism that may augment resistance phenotypes when coupled with upstream regulatory elements such as IS*Aba1* [31,32]. This regulatory plasticity underscores why the mere presence of intrinsic oxacillinase genes cannot be interpreted as a resistance determinant without considering genomic context.

Acquired oxacillinases exhibit far greater clinical relevance. Numerous molecular epidemiology studies confirm that *bla*OXA-23-like genes are strongly associated with CRAB lineages and frequently embedded within mobile genetic elements that facilitate horizontal dissemination [13,29,49]. The persistence of OXA-23-producing clones across hospital environments highlights their evolutionary fitness under antimicrobial selection pressure. Similarly, *bla*OXA-24/40-like genes, although regionally distributed, contribute significantly to endemic resistance patterns and long-term clonal stability [33,50].

In addition to class D β-lactamases, extended-spectrum β-lactamase (ESBL) genes such as *bla*TEM are widely reported in *A. baumannii* isolates and frequently coexist with carbapenemase determinants [1,17,51]. While ESBL genes alone do not confer carbapenem resistance, their co-occurrence with OXA-type enzymes contributes to multidrug-resistant phenotypes and reflects the cumulative acquisition of resistance modules. This layered resistance architecture is characteristic of high-risk clones and genomic resistance islands [37,39].

Metallo-β-lactamase genes, including *bla*VIM-like variants, are detected less frequently but possess disproportionate clinical importance due to their potent carbapenem-hydrolyzing capacity [1,34,52]. Epidemiological analyses suggest that MBL-producing *A. baumannii* isolates often arise within highly resistant genetic backgrounds, where coexistence with OXA enzymes and other resistance determinants exacerbates treatment limitations [52].

Aminoglycoside resistance genes further expand the adaptive landscape of *A. baumannii*. Genes encoding aminoglycoside-modifying enzymes (AMEs), particularly *aacC1* and related determinants, are commonly identified within integrons and resistance islands [8,12,35,53]. Their widespread distribution supports the frequent observation of aminoglycoside resistance phenotypes in MDR and XDR isolates. Importantly, co-localization of AME genes with carbapenem resistance determinants promotes co-selection and stabilizes multidrug resistance under antimicrobial pressure [28,36,54].

The epidemiological significance of resistance gene combinations warrants particular attention. Studies employing genome-scale analyses demonstrate that composite resistance regions, rather than isolated genes, drive the persistence and global spread of high-risk *A. baumannii* clones [37,39,55]. These genomic platforms enable simultaneous transmission of β-lactam, aminoglycoside, and other resistance determinants, thereby reinforcing phenotypic multidrug resistance.

Collectively, the reviewed evidence indicates that resistance gene prevalence, regulatory context, and genomic organization collectively determine both antimicrobial resistance phenotypes and the evolutionary success of circulating *A. baumannii* lineages. These factors provide a mechanistic basis for interpreting resistance surveillance data and contextualizing local resistance gene distributions within global epidemiological frameworks [1,13,56,57].

### 3.6. Global Trends and Evolutionary Dynamics of Resistance Determinants

Accumulating genomic and molecular epidemiology evidence indicates that antimicrobial resistance in *Acinetobacter baumannii* is shaped by dynamic evolutionary processes rather than isolated gene acquisition events. Contemporary surveillance studies consistently demonstrate that resistance determinants follow distinct dissemination trajectories governed by selective antibiotic pressure, mobilization potential of genetic platforms, and clonal expansion dynamics [1,13,29,56]. These observations support the concept that resistance evolution in *A. baumannii* is structured, non-random, and mechanistically constrained.

Among carbapenem resistance determinants, *bla*OXA-23-like genes remain the most globally stable and epidemiologically dominant. Multiple large-scale investigations describe persistent high prevalence of OXA-23-producing lineages across continents, suggesting strong evolutionary conservation and sustained adaptive advantage [13,50]. The remarkable persistence of this determinant has been mechanistically linked to its frequent association with transposons and genomic resistance islands, which collectively promote both vertical stability and horizontal dissemination [36,37]. Such genomic configurations enable resistant clones to persist over extended periods within hospital ecosystems.

In contrast, the global behavior of *bla*OXA-24/40-like genes appears more heterogeneous. Recent genomic studies reveal regionally concentrated expansion events, frequently associated with specific successful clonal lineages [33,50]. This divergence between OXA-23 global dominance and OXA-24/40 regional clustering highlights that resistance gene dissemination is strongly influenced by clonal background and local ecological conditions rather than universal selective forces.

To synthesize the principal evolutionary patterns governing resistance determinants in *A. baumannii*, the dominant mechanisms and dissemination behaviors are summarized in Table 2.

Insertion sequences represent one of the most influential evolutionary accelerators within *A. baumannii* genomes. IS*Aba1*-mediated promoter activation has been repeatedly implicated in modulating resistance gene expression, producing significant variability in phenotypic resistance levels without structural gene modification [31,32]. This regulatory plasticity provides a mechanistic explanation for heterogeneous resistance phenotypes observed among genetically similar isolates and contributes to adaptive flexibility under fluctuating antimicrobial pressure.

Resistance island-mediated aggregation further reinforces evolutionary robustness. *Aba*R and *Ab*GRI-type genomic islands function as composite reservoirs of multidrug resistance determinants and facilitate the co-selection of unrelated resistance mechanisms [36,39,55]. Comparative genomics analyses demonstrate ongoing structural diversification of these islands, suggesting continuous evolutionary remodeling driven by recombination and gene acquisition events [37,55]. Such mosaic genomic structures enhance adaptability and persistence of successful clonal lineages.

Importantly, global resistance dynamics cannot be separated from healthcare-associated ecological pressures. Antibiotic-intensive environments, particularly ICUs, act as evolutionary hotspots favoring the selection and stabilization of resistant genotypes [18,24,56,58]. The international dissemination of high-risk clones exemplifies the interplay between genomic adaptability and anthropogenic selective forces.

Major high-risk clonal lineages of *A. baumannii* are defined using multilocus sequence typing (MLST) schemes (Pasteur and Oxford), and the most clinically important are designated as Global Clones (GC). Global Clone 2 (GC2), corresponding to sequence type ST2 (Pasteur scheme) or ST208 (Oxford scheme), is the most frequently detected multidrug-resistant lineage worldwide and is disproportionately associated with carbapenem resistance and hospital outbreaks [13,29]. Global Clone 1 (GC1, ST1 Pasteur) also contributes significantly to global CRAB dissemination, albeit with greater geographic variation in prevalence. Emerging sequence types, including ST25, ST79, ST85, and regionally predominant clones in Asia, Europe, and the Middle East, are increasingly described in contemporary surveillance studies [29,56]. The epidemiological importance of these clonal lineages lies not only in their resistance gene content, but in their genomic platforms—particularly resistance islands and mobile elements—that enable stable co-carriage of multiple resistance determinants and facilitate long-term nosocomial persistence. Recognition of circulating sequence types is therefore essential for interpreting outbreak dynamics, tracking resistance gene dissemination, and designing surveillance strategies aligned with locally dominant clonal backgrounds.

Collectively, current evidence indicates that resistance determinants in *A. baumannii* follow multiple evolutionary trajectories: globally conserved dissemination (*bla*OXA-23-like), regionally adaptive persistence (*bla*OXA-24/40-like), regulatory amplification (IS*Aba1*-mediated effects), and genomic aggregation (resistance islands). Recognition of these structured evolutionary patterns is essential for interpreting resistance surveillance data and anticipating emerging resistance configurations [1,13,29,59,60,61,62,63,64].

## 4. Discussion

Antimicrobial resistance in *Acinetobacter baumannii* is best understood as an emergent systems-level phenotype arising from interactions among horizontally acquired resistance determinants, regulatory elements, genomic context, and healthcare-associated ecological pressures [1,13,29,56]. The evidence synthesized in this review highlights that neither phenotype nor genotype alone provides a complete resistance interpretation, and that clinically meaningful inference requires integration across multiple layers of data—particularly for carbapenems and aminoglycosides, where treatment decisions are most constrained [18,24,65,66,67].

A major interpretive axis in CRAB is the distinction between gene carriage and gene expression. While OXA-type carbapenemases dominate CRAB globally, the phenotypic magnitude of carbapenem resistance is frequently shaped by regulatory architecture, including insertion sequence–mediated promoter activity and gene amplification events [31,32]. This explains why isolates with similar carbapenemase repertoires can display heterogeneous MIC distributions and categorical susceptibility outcomes. Recent methodological syntheses emphasize that resistance prediction pipelines based solely on gene presence can misclassify borderline phenotypes when regulatory context is not captured [44,45,55,68]. This point is particularly important for surveillance systems that increasingly attempt genome-based inference as a replacement for phenotypic AST.

Conversely, carbapenem-resistant phenotypes without canonical carbapenemase genes remain biologically plausible and clinically relevant. Experimental and clinical studies support substantive contributions from RND efflux systems, permeability shifts, and adaptive regulations—mechanisms that can elevate carbapenem MICs independently or synergistically with β-lactamases [15,41,66]. These observations strengthen a central conclusion for clinical microbiology practice: phenotypic AST remains indispensable, and molecular panels should be framed as mechanistic complements rather than stand-alone decision tools [42,54,60].

For aminoglycosides, the literature supports a similar multi-layer model. AME genes are core determinants of resistance potential, yet phenotypic expression depends on enzyme–substrate specificity, gene dosage, and background resistance architecture [12,35,53]. Recent one-health and hospital ecology syntheses note that aminoglycoside resistance determinants often co-segregate with carbapenem resistance within mobile platforms, producing co-selection effects under ICU antibiotic pressure [56,65,68]. Clinically, this erodes the reliability of aminoglycosides as consistent “partner drugs” in CRAB combination regimens and reinforces the need for isolate-level testing and local antibiogram awareness [65,67,69].

A second major theme is the genomic organization and mobility of resistance determinants. Resistance islands (*Aba*R/*Ab*GRI) and related composite regions provide the structural basis for the persistence of MDR/XDR phenotypes by aggregating multi-class determinants and enabling clonal stabilization [36,39,55]. Recent genome-scale studies highlight that these islands are not static; rather, they are continuously remodeled through recombination and modular acquisition, yielding lineage-specific resistomes with distinct phenotypic signatures [55,68,70]. This dynamic architecture also explains why “same gene, different phenotype” remains common: genomic neighborhood effects and regulatory wiring matter as much as gene identity [31,32,45].

A third theme concerns methodological heterogeneity as a non-trivial source of apparent discordance. Breakpoint frameworks (EUCAST vs. CLSI), platform-specific variability, and interpretive rule updates can shift *susceptible* (S), *intermediate* (I), or *resistant* (R) categorization even when biology is stable [25,26,42,54]. Modern methodological reviews emphasize that cross-study comparisons must explicitly account for these factors; otherwise, temporal “trends” may reflect breakpoint drift rather than true epidemiologic change [42,54,71]. Similarly, WGS-based AMR prediction is limited by incomplete knowledge of regulatory mechanisms and under-characterized determinants, calling for cautious claims when translating resistome profiles into clinical susceptibility expectations [44,45,60,72].

Whole-genome sequencing and publicly accessible WGS databases are increasingly central to the analysis of antimicrobial resistance in *A. baumannii*. Online repositories such as NCBI’s National Database of Antibiotic Resistant Organisms (NDARO), the PATRIC/BV-BRC platform, PubMLST, and the CARD (Comprehensive Antibiotic Resistance Database) enable standardized annotation of resistance genes, tracking of sequence types, and large-scale comparative genomics across international isolate collections [57,60]. The ResFinder and AMRFinder tools, linked to curated databases, allow rapid prediction of resistance phenotypes from genome assemblies and facilitate benchmarking of genotype–phenotype concordance across studies [44,45]. Importantly, these platforms also support the surveillance of emerging resistance determinants and enable identification of novel gene variants that may not yet be detected by conventional PCR panels. However, the utility of WGS databases is contingent on data deposition practices, geographic representation of sequenced isolates, and the completeness of resistance gene annotations—all of which remain heterogeneous across participating institutions and countries. Encouraging routine WGS data submission and standardizing reporting frameworks would substantially enhance the capacity of global surveillance networks to monitor resistance evolution and support evidence-based antimicrobial stewardship [57,60,72].

Finally, the broader epidemiologic picture supports the view that CRAB is a healthcare-ecology problem, with ICU settings operating as evolutionary hotspots where antibiotic pressure, invasive devices, and transmission networks facilitate persistence and clonal expansion [18,24,65]. Contemporary clinical syntheses on treatment options further show that even when novel agents or combinations are considered, resistance evolution and within-host selection can rapidly undermine efficacy, particularly in high-burden units [67,73]. These realities argue for integrated interventions: robust infection prevention, stewardship aligned with local resistance ecology, and surveillance frameworks that combine phenotypic AST with targeted genomics [18,24,56,74].

In sum, the literature supports a multidimensional framework in which resistance in *A. baumannii* is driven by (i) acquired carbapenemases and AMEs; (ii) regulatory accelerators such as insertion sequences; (iii) genomic platforms that aggregate and stabilize multi-class resistance; and (iv) healthcare ecological pressures that select and disseminate high-risk clones [1,13,29,36]. This framework is essential for interpreting phenotype–genotype discordance, designing realistic diagnostic workflows, and aligning treatment and stewardship strategies with the evolutionary behavior of this pathogen [42,54,67,72].

Limitations of the Review. Several limitations inherent to the present review should be acknowledged. First, although a structured and transparent literature identification strategy was employed, this study was conducted as a narrative review rather than a formal systematic review with meta-analysis. Consequently, quantitative synthesis of resistance prevalence, pooled effect estimates, and statistical heterogeneity analysis were not performed. The conclusions presented therefore rely on qualitative integration of findings across heterogeneous study designs, geographic regions, and clinical settings.

Second, the included studies varied substantially in methodological approaches to antimicrobial susceptibility testing (AST), breakpoint interpretation (EUCAST vs. CLSI), molecular detection techniques, and genomic analysis pipelines. Such variability may influence reported resistance rates, genotype–phenotype concordance, and detection sensitivity for specific resistance determinants. Although these methodological differences were critically discussed, residual inter-study heterogeneity may limit direct comparability of findings.

Third, genome-based resistance prediction remains an evolving field. While WGS provides high-resolution insight into resistance determinants and their genetic context, current prediction models do not consistently account for regulatory effects, gene expression variability, epistatic interactions, and emerging or poorly characterized resistance mechanisms. Therefore, conclusions regarding genotype–phenotype relationships are constrained by the present state of genomic annotation and resistance databases.

Fourth, publication bias cannot be excluded. Studies reporting high resistance rates, outbreak events, or novel resistance mechanisms are more likely to be published than investigations demonstrating low prevalence or negative findings. This may overrepresent certain resistance determinants or geographic trends within the synthesized literature.

Finally, antimicrobial resistance in *Acinetobacter baumannii* is a rapidly evolving phenomenon. Breakpoint revisions, newly described resistance genes, emerging clones, and evolving therapeutic strategies may alter resistance interpretation over time. As a result, conclusions drawn in this review reflect the current evidence base within the defined publication window and should be interpreted in light of ongoing scientific and epidemiological developments.

Despite these limitations, the structured synthesis of phenotypic and genotypic evidence presented here provides a comprehensive framework for interpreting antimicrobial resistance dynamics in *A. baumannii* and for guiding integrated diagnostic and surveillance strategies.

## 5. Conclusions

Antimicrobial resistance in *Acinetobacter baumannii* represents a multidimensional phenomenon shaped by the interplay of acquired resistance genes, regulatory elements, genomic architecture, and healthcare-associated selective pressures. The accumulated evidence indicates that resistance phenotypes cannot be fully interpreted through gene detection alone, nor can phenotypic AST independently elucidate the underlying molecular mechanisms. Instead, resistance in *A. baumannii* emerges from a dynamic interaction between genetic potential and its functional expression within specific clinical and ecological contexts.

Carbapenem resistance is predominantly driven by OXA-type carbapenemases, yet variability in expression, insertion sequence-mediated regulation, and genomic context significantly modulate phenotypic outcomes. Similarly, aminoglycoside resistance frequently reflects the presence of aminoglycoside-modifying enzymes embedded within mobile genetic platforms, often co-selected alongside β-lactam resistance determinants. These findings reinforce that multidrug resistance in *A. baumannii* is rarely a single-gene event but rather a structured genomic configuration maintained by selective pressure and clonal dissemination.

The recurring observation of phenotype–genotype discordance underscores the necessity of integrative diagnostic frameworks. Molecular methods provide mechanistic resolution and epidemiological insight, whereas phenotypic AST remains indispensable for direct therapeutic decision-making. Optimal surveillance and clinical interpretation therefore require coordinated use of both approaches, accompanied by transparent reporting of methodological standards and breakpoint systems.

From an epidemiological perspective, resistance evolution in *A. baumannii* and continued genomic surveillance to detect emerging resistance architectures. In this context, publicly accessible WGS databases—including CARD, NDARO, PubMLST, and BV-BRC—represent essential infrastructure for tracking resistance gene dissemination, monitoring clonal dynamics, and enabling cross-institutional benchmarking of genotype–phenotype concordance. Integrating routine WGS data deposition into clinical and surveillance workflows will be critical for translating genomic insights into actionable public health responses.

In summary, effective management of antimicrobial resistance in *Acinetobacter baumannii* demands an integrated, systems-level perspective that bridges phenotypic data, genomic determinants, regulatory mechanisms, and clinical context. Such an approach provides the most reliable foundation for translating laboratory findings into meaningful epidemiological surveillance and optimized patient care.

## Figures and Tables

**Table 1:** Mechanisms underlying phenotype–genotype concordance and discordance in *Acinetobacter baumannii*.

Scenario	Observed Phenotype	Genotypic Findings	Mechanistic Basis	Representative Sources
Classical concordance	Carbapenem resistance	*bla*OXA-23/OXA-24/40 detected	Carbapenemase-mediated β-lactam hydrolysis	[7,13,31,33]
Expression-driven variability	Variable carbapenem MICs	Carbapenemase gene present	Regulatory effects (IS*Aba1*, expression modulation)	[31,32,40]
Genotype without phenotype	Carbapenem susceptibility	Carbapenemase gene detected	Low expression/regulatory inactivity	[32,40,42]
Phenotype without target gene	Carbapenem resistance	No carbapenemase gene detected	Efflux systems, membrane alterations	[15,41]
Aminoglycoside discordance	Partial susceptibility/resistance	AME genes detected	Enzyme specificity, expression variability	[12,35,43]
Clone-specific resistance patterns	Stable MDR/XDR profiles	AbaR/AbGRI islands present	Co-localized resistance determinants	[37,39]
Technical discordance	AST variability	Stable genotype	Breakpoint/method differences	[25,26,42,46,47,48]

**Table 2:** Evolutionary and epidemiological patterns of major resistance determinants in *Acinetobacter baumannii*.

Determinant/Mechanism	Observed Global Pattern	Evolutionary Drivers	Epidemiological Implication	Key Sources
*bla*OXA-23-like	Globally dominant, stable	Transposons, resistance islands	Persistent CRAB lineages worldwide	[13,36,50]
*bla*OXA-24/40-like	Regionally clustered	Clonal background, local selection	Local endemic resistance patterns	[33,50]
IS*Aba1* regulatory effects	Cross-lineage occurrence	Promoter activation, gene amplification	Variable resistance magnitude	[31,32]
*Aba*R/*Ab*GRI islands	Highly diverse, mosaic	Recombination, gene aggregation	Co-selection of MDR/XDR traits	[36,37,55]
High-risk clones	Globally disseminated	Clonal expansion, selection pressure	Hospital persistence, outbreaks	[13,56]

## Data Availability

No new data were created or analyzed in this study. Data sharing is not applicable to this article.

## Abbreviations

The following abbreviations are used in this manuscript:

AMR	Antimicrobial resistance
AME	Aminoglycoside-modifying enzyme
AST	Antimicrobial susceptibility testing
CLSI	Clinical and Laboratory Standards Institute
ESBL	Extended-spectrum β-lactamase
EUCAST	European Committee on Antimicrobial Susceptibility Testing
HAI	Healthcare-associated infection
MBL	Metallo-β-lactamase
MDR	Multidrug-resistant
MIC	Minimum inhibitory concentration
OXA	Oxacillinase-type β-lactamase
PCR	Polymerase chain reaction
RND	Resistance-nodulation-division
WGS	Whole-genome sequencing
WHO	World Health Organization
XDR	Extensively drug-resistant
